# Supplementary material for: Oxidative Stress-Induced DNA Damage and Apoptosis in Clove Buds-Treated MCF-7 Cells
Source: Biomolecules. 2020 Jan 14;10(1):139. doi: 10.3390/biom10010139 (PMC7022383; doi:10.3390/biom10010139)
Supplement: Supplementary file 1 [file biomolecules-10-00139-s001.pdf]

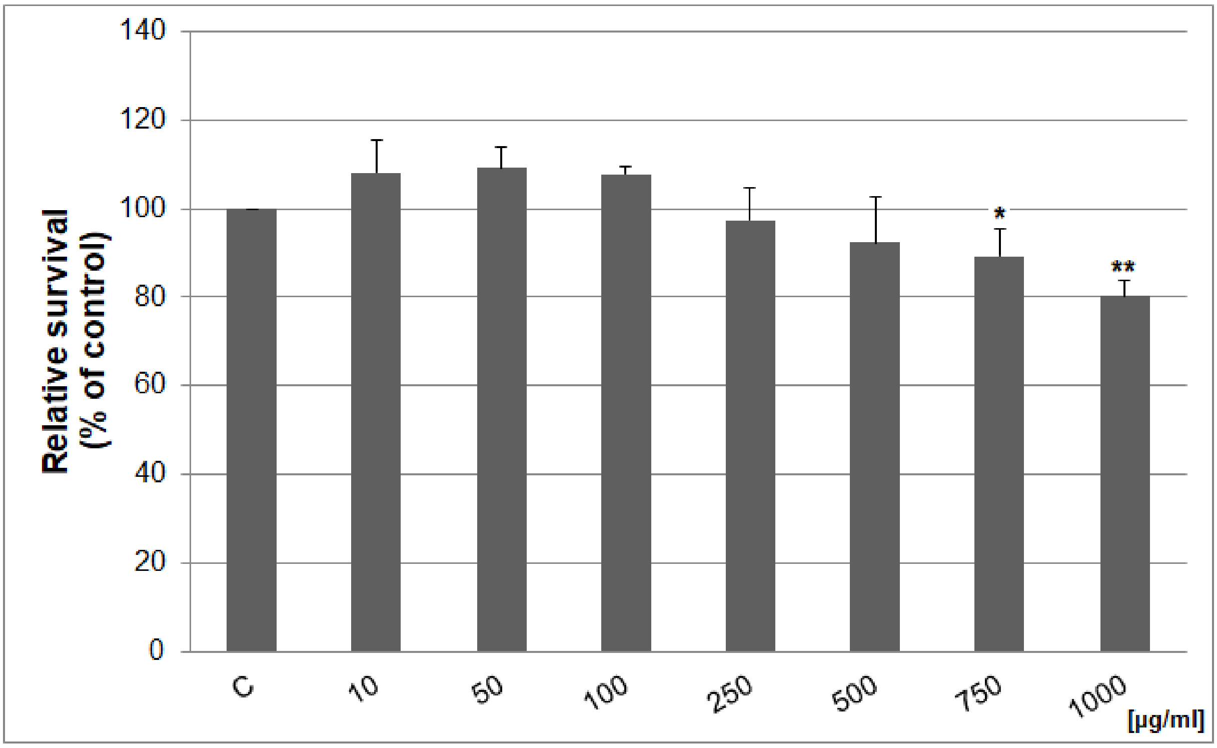

Figure S1. MCF-10A cells proliferation after CBE treatment. Data were obtained from three independent measurements. Significantly different * $P < 0.05$, ** $P < 0.01$ versus untreated cells (control).
